# The Role of Adipose Tissue and Obesity in Causing Treatment Resistance of Acute Lymphoblastic Leukemia

**DOI:** 10.3389/fped.2014.00053

**Published:** 2014-06-05

**Authors:** Xia Sheng, Steven D. Mittelman

**Affiliations:** ^1^Diabetes and Obesity Program, Center for Endocrinology, Diabetes and Metabolism, Children’s Hospital Los Angeles, Los Angeles, CA, USA; ^2^Keck School of Medicine, University of Southern California, Los Angeles, CA, USA; ^3^Department of Pediatrics, Keck School of Medicine, University of Southern California, Los Angeles, CA, USA; ^4^Department of Physiology and Biophysics, Keck School of Medicine, University of Southern California, Los Angeles, CA, USA

**Keywords:** adipocytes, tumor microenvironments, leukemia, pharmacokinetics, obesity, lipolysis, apoptosis, drug resistance

## Abstract

Obesity is responsible for ~90,000 cancer deaths/year, increasing cancer incidence and impairing its treatment. Obesity has also been shown to impact hematological malignancies, through as yet unknown mechanisms. Adipocytes are present in bone marrow and the microenvironments of many types of cancer, and have been found to promote cancer cell survival. In this review, we explore several ways in which obesity might cause leukemia treatment resistance. Obese patients may be at a treatment disadvantage due to altered pharmacokinetics of chemotherapy and dosage “capping” based on ideal body weight. The adipose tissue provides fuel to cancer cells in the form of amino acids and free fatty acids. Adipocytes have been shown to cause cancer cells to resist chemotherapy-induced apoptosis. In addition, obese adipose tissue is phenotypically altered, producing a milieu of pro-inflammatory adipokines and cytokines, some of which have been linked to cancer progression. Given the prevalence of obesity, understanding its role and adipose tissue in acute lymphoblastic leukemia treatment is necessary for evaluating current treatment regimen and revealing new therapeutic targets.

## Introduction

Obesity is a serious health problem in both adults and children. Data from the National Health and Nutrition Examination Survey of 2009–2010 revealed that more than 35% of adults and almost 17% of youth were obese^1^ in the United States (1). Another one-third of adults and one-sixth of children are overweight, meaning that overall most adults and about a third of children have unhealthy weight. The estimated annual medical cost of obesity in the U.S. was $209.7 billion, or $2741 for each obese person (2). Obesity is associated with a variety of health conditions, including type II diabetes (3), cardiovascular diseases, hypertension, osteoarthritis, and cancer (4). However, the mechanism(s) whereby obesity increases cancer incidence and mortality are largely unknown.

There is now increased understanding that the cancer microenvironment plays an important role in spread, metastasis, and treatment response. This microenvironment consists of cancer cells, normal cells, and the intracellular matrix and signals surrounding them. In solid tumors, cancer cells interact with several types of host cells including fibroblasts, macrophages, lymphocytes, endothelial cells, and adipocytes. Through a complex set of interactions, which are not completely understood, these host cells are recruited to transform the local environment into a hospitable niche, improving access to nutrients, protecting from immune surveillance, and providing growth factors and survival signals (5). Together, these changes allow the cancer to survive, proliferate, and metastasize (6). Given their mobile nature and propensity to travel throughout the body, leukemia cells are exposed to several microenvironments, including the bone marrow, spleen, lymphatic system, the intravascular environment, and various extramedullary tissues. Understanding the roles of these various microenvironments is important for further improving treatment outcome.

**Figure 1:**
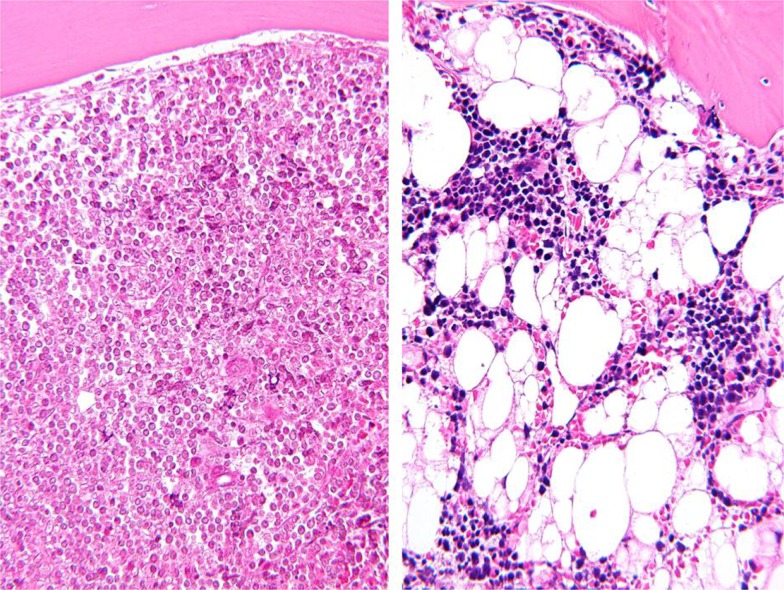
**Representative bone marrow biopsies from a patient with childhood ALL before (left) and after (right) induction chemotherapy**. Picture taken by Hiro Shimada (Wright-Giemsa stain, magnified 200×).

Adipose tissue and adipocytes have been shown to play an important role in supporting progression of several types of cancer. Bone marrow, a major site of metastasis for solid tumors and an important microenvironment for hematological malignancies, is also rich in adipocytes. In fact, after induction chemotherapy for acute lymphoblastic leukemia (ALL), adipocytes can represent the primary cellular component of bone marrow (Figure 1). Given the effects of obesity on cancer prognosis, we and others have investigated whether these fat cells may contribute to treatment resistance in leukemias and other cancers. The goal of this mini review is to summarize our current understanding on how obesity/adipose tissue contributes to leukemia relapse and drug resistance.

## Does Obesity Directly Increase Leukemia Mortality?

Obesity is strongly associated with increased cancer incidence and mortality. A large prospective study published in 2003 by Eugenia Calle and colleagues was one of the first to show that increased BMI is associated with increased mortality from many types of cancer (7). The study found an overall 52% increased mortality in men with BMI ≥35, and an 88% increase in women of the same BMI category. While much of this increase in mortality can be attributed to increased cancer incidence, obesity has also been shown to associate with a poorer prognosis in a variety of cancers.

Several studies have found an increased risk of developing leukemia among the obese (8–11). In a meta-analysis of cohort studies, Larsson and Wolk found that excess body weight is associated with an increased risk of developing all four major subtypes of hematologic malignancies [ALL, acute myelogenous leukemia (AML), chronic lymphocytic leukemia (CLL), and chronic myelogenous leukemia (CML)] (12). They estimated that each 5 kg/m^2^ of increased BMI is associated with a 13% increase risk of developing leukemia. Others have shown that obesity increases the risk of developing Hodgkin’s lymphoma, non-Hodgkin’s lymphoma, and multiple myeloma (13–15).

The association between obesity and leukemia prognosis has also been examined in many studies, with some detecting an effect of obesity to worsen prognosis, and others not (Table 1). Two of the studies acknowledged that their failure to detect an association between BMI and ALL outcome may have been due to small sample size (16, 17). Interestingly, the risk estimates of overall survival and event-free survival from both studies showed a trend of worsened outcome in the overweight/obese patients. The largest study was done by the CCG, and included over 5000 children (18). This study found that obesity was associated with a significantly increased risk of relapse, particularly in children over 10 years of age (considered high risk). This latter caveat is consistent with the findings of a relatively small study of 337 patients from the UKALL X treatment trial, which included only standard risk patients, and concluded that overweight or obesity at diagnosis was unlikely to impair prognosis (20). Thus, it appears that obesity can impair ALL outcome at least in high risk, older patients.

**Table 1:** **Retrospective studies examining the association between overweight/obesity and ALL outcome (EFS, event free survival; HR, hazard ratio)**.

Study	Population (age)	Cooperative group/regimen	No	Results	Conclusion
Baillargeon (16)	Predominantly Hispanic B-precursor ALL patients (2–9 years)	South Texas pediatric minority based community clinical oncology program/pediatric oncology group legacy protocols	241	HR of obesity on EFS = 1.09 (0.54–2.20)	“No association between obesity and survival”
	Predominantly Hispanic B-precursor ALL patients (10–18 years)	South Texas pediatric minority based community clinical oncology program/pediatric oncology group legacy protocols	81	HR of obesity on EFS = 1.48 (0.73–3.01)	
Hijiya (17)	Predominantly white ALL	St Jude total therapy studies/total XII, XIIIA, XIIIB, and XIV	621	5 year EFS: obese = 72.7 ± 5.9%, normal weight = 78.7 ± 2.1%, *p* = 0.722	“No association between BMI and outcome or toxicity in children with ALL”
	Subset ≥10 years	St Jude total therapy studies/total XII, XIIIA, XIIIB, and XIV	185	*p* = 0.103 For effect of obesity on EFS	“Relatively small number of patients and the use of different treatments compared to those of the CCG study”
Butturini (18)	Predominantly white newly diagnosed ALL	CCG1881 CCG1922 CCG1891 CCG1882 CCG1901	4260	HR of obesity on events 1.26 (1.03–1.55), *p* = 0.02	“In this study, obesity seems to be one of the main determinants of relapse in … patients diagnosed with ALL after their 10th birthday …”
	Predominantly white newly diagnosed ALL (≥10 years)	CCG1882 CCG1901	1003	HR of obesity on events 1.48 (1.07–2.03), *p* = 0.01	
	Verification cohort (≥10 years)	CCG1961	1160	Obese and ≥10 years old: 1.42 (1.03–1.96) *p* = 0.032	
Gelelete (19)	Children with ALL (mainly <10 years)	IPPMG/UFRJ/Berlin–Frankfurt–Munich protocols	181	HR of overweight and obese on EFS 1.92 (1.42–2.6) *p* = 0.031	“… Overweight or obesity at diagnosis was an independent prognostic factor to the 5-years EFS in children with ALL …”
Aldhafiri (20)	UK national cohort excluding high risk and low risk (2–15 years)	UKALL X treatment trial	337	Relapse rate in overweight/obese = 36.2%, healthy weight = 36.6%	“No evidence that being overweight/obese at diagnosis impairs prognosis in childhood ALL in the UK”

Since obesity is associated with many potential confounding socioeconomic, genetic, behavioral, environmental, and treatment factors, it is difficult to determine whether it is causally related to leukemia development and prognosis, or rather associated with some other prognostic variable. For example, a genetic or environmental factor could in theory predispose some children to develop both obesity and leukemia. Some studies have suggested that obese patients may receive suboptimal care due to difficulty performing basic physical and radiologic assessments, which could contribute to poorer outcome (21, 22). While these possibilities are difficult to tease apart in patients, Yun et al. performed a study, which showed that diet-induced obesity directly accelerates ALL progression in two mouse models (23). This was the first indication that the observed associations between obesity and leukemia incidence are likely to be directly related to the biological effects of obesity *per se*. However, the authors were not able to identify any specific mechanisms responsible for this effect, though several hormones, such as insulin, leptin, and IL-6, were elevated in the obese mice.

## Adipose Tissue

Adipose tissue is a major body constituent. Healthy men and women are composed of ~8–19 and 21–33% fat, respectively (24). Morbidly obese individuals can have well over 40% body fat. By volume, adipocytes are the major cell type found in adipose tissue. However, preadipocytes, fibroblasts, endothelial cells, immune cells, adipose stem cells, and adipose tissue macrophages are also found in adipose tissue (25–28). Particularly in obese individuals, these other cell types can account for more than half of the number of cells in adipose tissue.

Traditionally, adipose tissue has been recognized as primarily a site for fuel storage. However, since the discovery of leptin by Zhang et al. in 1994, it is now well-established that adipose tissue is an active endocrine organ (29). There are now more than 50 identified adipokines (i.e., cytokines secreted primarily by adipose tissue) (30). These adipokines regulate appetite, energy expenditure, immune function, growth, and metabolism of other tissues. Many adipokines (such as leptin) and other cytokines secreted by adipose tissue (such as IGF-1), have been linked to cancer pathogenesis.

Numerous immune cells are found in normal adipose tissue, including T and B lymphocytes, NK cells, NKT cells, mast cells, macrophages, and neutrophils. Adipose tissue inflammation likely contributes to many of the negative sequelae of obesity, including diabetes, heart disease, and possibly cancer (31). Obesity is associated with an increased number of lymphocytes in adipose tissue in mice (12). These lymphocytes are believed to interact with adipocytes and adipose tissue macrophages, and may play a role in obesity-induced insulin resistance and diabetes (32).

## Adipocytes in the Cancer Microenvironment

Several cancers occur in close proximity to adipose tissue. Breast, colon, pancreas, ovary, uterus, and liver are all surrounded by and/or infiltrated by adipose tissue. Extension of these cancer types outside of their originating organ often takes them into direct contact with adipose tissue. Furthermore, adipocytes are found in the bone marrow, a common site for solid tumor metastasis (33), and an important microenvironment for many hematologic malignancies (34). Bone marrow adiposity is not only affected by obesity (35), but has recently been shown to be influenced by ALL treatment (36). Vicente López et al. isolated mesenchymal stem cells (MSCs) from bone marrow aspirates of ALL patients at various timepoints: diagnosis, during therapy, and after therapy. ALL-MSC from treated patients showed an increased adipogenic differentiation potential, including a higher expression of adipogenic genes (CEBP and PPARgamma), compared to healthy MSC (36).

Given the fact that lymphocytes infiltrate adipose tissue, it is not surprising that adipocytes attract preB leukemia cells as well. We found that ALL cells were present in adipose tissue of mice, which developed progressive leukemia despite vincristine treatment (37). In addition, syngeneic ALL cells implanted into mice by a retro-orbital injection infiltrated adipose tissue within 10 days, to a similar degree as other more classic sites for ALL, such as spleen and liver (38). ALL migration toward adipocytes is mediated by adipocyte secretion of stromal cell-derived factor 1 alpha (SDF-1α or CXCL12). While obesity was not associated with increased serum levels of SDF-1α, obese mice had a significantly higher burden of leukemia cells in visceral fat compared to control mice (38). Adipocytes have been shown by others to facilitate leukemia bone marrow engraftment via secretion of SDF-1α and leptin (39). Adipocytes also promote invasion of a number of other cancer types, including ovarian, gastric, breast, and colon (40, 41).

Some cancer cells directly interact with adipocytes and induce a phenotypic change, turning them into “cancer associated adipocytes” (CAA) (41), though this has not to our knowledge been observed in leukemia. Reprogramed adipocytes provide growth factors and fuel to cancer cells, promoting metastasis, and sustaining uncontrolled growth. Ribeiro et al. have shown that peri-prostatic adipose tissue explants from overweight/obese prostate cancer patients had significantly elevated MMP9 activity, which is correlated with disease progression and metastasis (42). In breast cancer, adipocyte-derived collagen IV (43) and endotrophin (44) have been shown to promote tumor progression.

## Adipose Tissue Provides Fuel for ALL Cells

One of the hallmarks of cancer is rapid proliferation, which requires a high amount of energy and nutrient building blocks. A major function of adipocytes is energy storage in the form of triglycerides, which can be broken down into glycerol and free fatty acids (FFA) and released in a process termed lipolysis. Therefore, it is possible that adipocytes provide FFA to cancer cells, facilitating their energy demands and contributing to their synthesis of lipid moieties such as phospholipid membranes and signaling molecules. Indeed, many cancer cell types have been shown to have lipid droplets, which may represent important energy stores (40, 45, 46).

There is recent evidence of adipocytes providing FFA as fuel source to leukemia cells. A recent paper by Tung et al. found that FFAs could support CLL metabolism and cause resistance to glucocorticoid-mediated cytotoxicity (47). They showed both adipocyte-conditioned media (ACM) and heat-inactivated ACM were able to reduce dexamethasone-induced CLL cell death, suggesting the factors in ACM to be lipids. However, there was no evidence of direct transfer of lipids from adipocytes to CLL. Tucci et al. have found evidence that ALL cells stimulate adipocyte lipolysis and use adipocyte-derived FFAs to supplement *de novo* lipogenesis and proliferation (48). Other cancer cell types, such as breast and ovarian cancer, have also been shown to induce lipolysis and dedifferentiation of nearby adipocytes (31, 40, 41).

Adipocytes also produce amino acids. Adipose tissue is a major source of glutamine, an important fuel for cancer cells, and a building block needed for synthesis of nucleic acids and proteins. l-asparaginase is used in ALL treatment because leukemic lymphoblasts are exquisitely sensitive to the depletion of exogenous asparagine and glutamine (49, 50). The drug hydrolyzes asparagine and glutamine to aspartic acid and glutamic acid, respectively. A recent paper by Ehsanipour et al. reported that adipocytes protect leukemia cells from l-asparaginase treatment by producing asparagine and glutamine (51). The authors treated leukemic diet-induced obese (DIO) and normal weight C57Bl/6 mice with the drug proportional to body weight and found significantly shortened survival in the DIO mice. An increase in both glutamine synthetase and adipocyte number was observed in bone marrow biopsy specimens from adolescent leukemia patients after induction treatment. Adipocyte protection of ALL cells against l-asparaginase was blocked by pretreatment with an inhibitor of glutamine synthetase. Since leukemia cells can infiltrate adipose tissue, it is possible that high local levels of these amino acids protects ALL cells from l-asparaginase.

## Adipose Tissue Alters Chemotherapy Pharmacokinetics

Obesity influences many aspects of drug pharmacokinetics (PK). For lipid-soluble drugs, obesity increases the volume of distribution by accumulation of drug in the excess adipose tissue (52–54). Obesity is also associated with increased alpha 1-acid glycoproteins, which could increase the binding of basic drugs in plasma (53). Hepatic and renal drug clearance could be altered in obesity due to increased activity of cytochrome P450 2E1 and increased glomerular filtration and tubular secretion (55, 56). This in turn could alter the PK of water-soluble drugs, since they can be readily excreted from the kidneys (57). Since the overall exposure to a drug depends on both the volume of distribution and the clearance, obesity could be associated with both toxicity and impaired efficacy of different medications. While there have been no studies of which we are aware examining the effects of weight status on PK in children with leukemia, a retrospective study by Hijiya et al. reported no difference in mean systemic clearance and intracellular levels of thioguanine nucleotides and methotrexate polyglutamates among four BMI groups. (17).

Vincristine, a potent anti-microtubule agent, is a key drug used in childhood ALL combination chemotherapy, as well as treatment of many other cancers. In pediatric and adult leukemia patients, vincristine is dosed proportional to body surface area, and this dose is generally “capped” at 1 square meter. We found that DIO mice implanted with syngeneic leukemia cells exhibited poorer survival compared to control mice, despite vincristine treatment being dosed proportional to body weight (37). However, there have been few PK studies examining the impact of obesity on chemotherapy treatments. Given that vincristine is a lipophilic agent, it may be sequestered in adipose tissue and thus have altered tissue distribution in obese individuals. We explored this idea using injections of tritiated vincristine in obese and control mice (58). Upon a single intravenous injection of vincristine proportional to body weight, blood and tissue levels of the drug were measured at different time points up to 24 h. Over the initial 24 h, blood vincristine concentrations were higher in the obese mice than the control, while tissue concentrations were comparable in spleen, liver, brown fat, and bone marrow. However, by 3 h, there was significantly higher vincristine in the white adipose tissue of obese mice than that of control mice. PK modeling showed that overall exposure of ALL cells to vincristine is impaired by obesity, and this may be exacerbated when the drug is dosed/body surface area and is capped.

In 2012, the American Society of Clinical Oncology published recommendations for appropriate cytotoxic chemotherapy dosing for obese adult cancer patients (59). The ASCO assembled a panel of experts to conduct a systematic review of studies published between 1996 and 2010. They reported that up to 40% of obese patients received reduced chemotherapy doses that were not based on actually body weight. Meanwhile, they found no evidence that short- or long-term toxicity was increased among obese patients receiving full weight-based doses. The panel thus recommends that “full weight-based chemotherapy doses be used in the treatment of the obese patient with cancer, particularly when the goal of treatment is cure.”

## Adipose Tissue Prevents Leukemia Apoptosis

There is growing evidence that adipocytes and other cells found in adipose tissue provide survival advantages to cancer cells in the face of chemotherapy. Using an *in vitro* TransWell co-culture system, Behan et al. have shown that adipocytes protect ALL cells from vincristine, nilotinib, daunorubicin, and dexamethasone (37). The authors found that adipocyte protection of ALL cells from vincristine was associated with upregulation of Bcl-2 and Pim-2 (both pro-survival signals in the intrinsic apoptosis pathway), and an increased phosphorylation of Bad (indicating inactivation of the pro-apoptotic protein). Beaulieu et al. reported that leptin from adipocytes can reverse pro-apoptotic and antiproliferative effects of alpha-linolenic acids in BCR-ABL chronic myeloid leukemia cells (60). In a separate study, MSC-derived adipocytes protected acute promyelocytic leukemia cells from apoptosis induced by serum starvation or by doxorubicin, possibly partially by leptin (61). Adipocytes have also been reported to cause chronic lymphoblastic leukemia cells to become resistant to dexamethasone by providing lipid factors (47).

A series of experiments published by Iyengar et al. demonstrated that adipocytes could induce both replicative potential and evasion of apoptosis in breast cancer cells (62). An upregulation of A20 and NFkB, both anti-apoptotic signals, was seen in MCF-7 breast cancer cells treated with ACM, suggesting a direct effect of adipokines on the cancer cells.

One of the adipokines that is elevated in obesity is leptin, which has been shown to promote the growth of several types of cancer. Adipose tissue is the main source of leptin secretion; however, normal and malignant breast tissue has been reported to also secrete leptin. Interestingly, high leptin and low adiponectin have been reported in children with ALL compared to age, sex, and BMI matched healthy controls (63). These findings are consistent with the evidence that leptin has proliferative effects on hematopoietic cells (64, 65), while adiponectin has a protective role against carcinogenesis (66–68). Leptin receptor mRNA has been detected in several myeloid and lymphoid leukemic cell lines, including some newly diagnosed cases of AML, ALL, and CML during blast crisis (69, 70). Leptin has been shown to be involved in the pathogenesis of breast and prostate cancers as well (71, 72).

Not only do adipocytes play an important role in the tumor microenvironment, adipose tissue stromal cells (ASC) have been shown to be recruited to tumors and enhance tumor vascularization, and promote survival and proliferation of the tumor cells (73, 74). These ASC have many features of bone marrow-derived mesenchymal stromal cells (75), including cell surface marker expression (76), plastic adherence (77), and the capacity to differentiate into osteoblasts, chondrocytes, and adipocytes (78). While ASCs have not been studied in the context of leukemia, they have been shown to promote the growth of human endometrial adenocarcinoma xenografts in immunodeficient nude mice, likely due to increasing vascularization (79).

## Potential Therapeutic Targets toward Drug Resistance Associated with Obesity

With the prevalence of obesity and the mounting evidence of increased adiposity as a risk factor for cancer incidence and mortality, it is important to understand the adipocyte-cancer interaction and expand traditional therapies to address obesity. More than two decades ago, Shields et al. showed that chronic energy-intake restriction (CEIR) has profound effects to delay lymphoma development in AKR mice (80). As a more practical strategy than CEIR, short-term fasting has been demonstrated to provide differential stress resistance to chemotherapy, where normal cells are protected while cancerous cells remain sensitive. There is evidence that fasting is not only safe, but also effective in reducing common side effects associated with chemotherapy (81).

There are a number of clinically available metabolic agents targeting adipocyte differentiation, insulin resistance, and inflammation that have been shown to have anti-cancer effects. One of such agents is metformin, which is clinically used to treat type 2 diabetes mellitus. While metformin has not been testing in leukemia patients, analysis of a retrospective cohort of 363 women diagnosed with endometrial cancer and diabetes mellitus analysis demonstrated that metformin use was associated with prolonged recurrence-free survival time (82).

Since adipocytes may be providing cancer cells with FFA as a fuel source, inhibition of lipolysis, and FFA efflux from adipocytes or blocking of cancer cell FFA oxidation could represent therapeutic targets. Inhibition of FFA oxidation, such as with etomoxir, has been shown to impair a variety of hematological malignancies, including myeloma cell (83), AML (84), CLL (85), and mantle cell lymphoma (86). Since cancer cells, such as ovarian cancer, increase their FFA oxidation rate when co-cultured with adipocytes (40), targeting this pathway may be particularly beneficial in obese patients.

## Conclusion and Perspective

Over the past two decades, there has been ground-breaking research in the field of adipocyte biology, leading to a reassessment of the role of adipose tissue in man. There has also been mounting epidemiological evidence of the important effect of obesity on cancer incidence and mortality. Adipocytes play an active role in the tumor microenvironment in many types of cancer, including those that have strong associations with obesity. With obesity rates reaching unprecedented levels worldwide and the alarming rise in childhood obesity, teasing apart the mechanisms linking obesity, and cancer represents a crucial step in the quest to improve overall cancer survival.

## Conflict of Interest Statement

The authors declare that the research was conducted in the absence of any commercial or financial relationships that could be construed as a potential conflict of interest.

## Footnotes

^1^Obesity is defined by the Center for Disease Control as a body mass index (BMI) ≥30 kg/m2 in adults or ≥95th percentile in children. Overweight is considered a BMI ≥25 but <30 kg/m2 in adults, and ≥85th but <95th percentile in children.
